# Chemotherapy for intracranial ependymoma in adults

**DOI:** 10.1186/s12885-016-2323-0

**Published:** 2016-04-23

**Authors:** Dorothee Gramatzki, Patrick Roth, Jörg Felsberg, Silvia Hofer, Elisabeth J. Rushing, Bettina Hentschel, Manfred Westphal, Dietmar Krex, Matthias Simon, Oliver Schnell, Wolfgang Wick, Guido Reifenberger, Michael Weller

**Affiliations:** Department of Neurology, University Hospital Zurich, Frauenklinikstrasse 26, 8091 Zurich, Switzerland; Department of Neuropathology, Heinrich-Heine-University, Moorenstrasse 5, 40225 Düsseldorf, Germany; German Cancer Consortium (DKTK), partner site Essen/Düsseldorf, German Cancer Research Center (DKFZ), Im Neuenheimer Feld 280, 69121 Heidelberg, Germany; Department of Oncology, University Hospital Zurich, Rämistrasse 100, 8091 Zurich, Switzerland; Department of Neuropathology, University Hospital Zurich, Schmelzbergstrasse 12, 8091 Zurich, Switzerland; Institute for Medical Informatics, Statistics and Epidemiology, University of Leipzig, Härtelstrasse 16-18, 04107 Leipzig, Germany; Department of Neurosurgery, University Medical Center Hamburg-Eppendorf, Martinistrasse 52, 20251 Hamburg, Germany; Department of Neurosurgery, Technical University Dresden, Fetscherstrasse 74, 01307 Dresden, Germany; Department of Neurosurgery, University of Bonn Medical School, Sigmund-Freud-Strasse 25, 53127 Bonn, Germany; Department of Neurosurgery, Ludwig Maximilian University Munich, Marchionistrasse 15, 81377 Munich, Germany; Clinical Cooperation Unit Neurooncology, German Cancer Consortium (DKTK), German Cancer Research Center (DKFZ), and Neurology Clinic and National Center for Tumor Diseases, University Hospital Heidelberg, Im Neuenheimer Feld 672, 69120 Heidelberg, Germany; Department of General Neurology, University Hospital Tübingen, Hoppe-Seyler-Strasse 3, 72076 Tübingen, Germany

**Keywords:** Adults, Chemotherapy, Intracranial ependymoma, Overall survival, Progression-free survival

## Abstract

**Background:**

Ependymal tumors in adults are rare, accounting for less than 4 % of primary tumors of the central nervous system in this age group. The low prevalence of intracranial ependymoma in adults limits the ability to perform clinical trials. Therefore, treatment decisions are based on small, mostly retrospective studies and the role of chemotherapy has remained unclear.

**Methods:**

We performed a retrospective study on 17 adult patients diagnosed with intracranial World Health Organisation grade II or III ependymoma, who were treated with chemotherapy at any time during the disease course. Benefit from chemotherapy was estimated by applying Macdonald criteria. Progression-free (PFS) and overall survival (OS) were calculated from start of chemotherapy, using the Kaplan-Meier method.

**Results:**

Eleven patients had supratentorial and 6 infratentorial tumors. Ten patients were treated with temozolomide (TMZ), 3 with procarbazine/lomustine/vincristine (PCV), 3 with platinum-based chemotherapy and 1 patient received epirubicin/ifosfamide. Response rates were as follows: TMZ 8/10 stable disease; PCV 3/3 stable disease; platinum-based chemotherapy 1/3 partial response; epirubicin/ifosfamide 1/1 complete response. PFS rates at 6, 12 and 24 months were 52.9, 35.3 and 23.5 %. OS rates at 6, 12 and 24 months were 82.4, 82.4 and 70.1 %. There was no indication for a favourable prognostic role of *O*^*6*^*-methylguanyl-DNA-methyltransferase* (*MGMT*) promoter methylation which was detected in 3/12 investigated tumors.

**Conclusions:**

Survival outcomes in response to chemotherapy in adult intracranial ependymoma patients vary substantially, but individual patients may respond to any kind of chemotherapy. There were too few patients to compare survival data between chemotherapeutic subgroups.

**Electronic supplementary material:**

The online version of this article (doi:10.1186/s12885-016-2323-0) contains supplementary material, which is available to authorized users.

## Background

Ependymomas are a histologically, biologically and clinically heterogenous group of glial tumors that show histological features of ependymal differentiation and preferentially are located in the cerebral ventricles or the spinal cord. In total, ependymomas account for 6.8 % of all gliomas, with the relative frequency being higher in children compared to adults [1]. In adults, ependymomas are less than 4 % of primary central nervous system tumors [2] and are found more often in spinal (46 %) than infra- (35 %) or supratentorial (19 %) locations [3]. These tumors are classified by the World Health Organisation (WHO) into 3 grades [4]. The prognostic relevance of the histopathological distinction between WHO grade II versus WHO grade III ependymomas has remained controversial [2, 5]. In comparison to WHO grade II ependymomas, WHO grade III ependymomas are associated with increased risk of treatment failure [3].

The low prevalence of intracranial ependymoma in adults limits opportunities to perform large clinical trials. Thus, treatment recommendations are based on small, mostly retrospective studies. Surgical resection is the most important therapeutic intervention for intracranial ependymomas [2]. Extent of resection has been associated with increased progression-free survival (PFS) and overall survival (OS) in most series [3, 6–8]. Adjuvant radiotherapy (RT) is recommended for patients diagnosed with anaplastic (WHO grade III) ependymoma [2, 9], whereas the role of RT in patients with WHO grade II ependymoma is discussed controversially. While resection followed by irradiation as first-line therapy is considered for posterior fossa ependymoma [10], RT for supratentorial ependymoma is commonly used only when surgical resection has been incomplete [2, 3, 11]. The role of chemotherapy (CT) for intracranial ependymoma in adults remains unclear. A variety of chemotherapeutic drugs has been investigated in the past, mostly in retrospective studies or case reports. Selected case reports demonstrate response to temozolomide (TMZ) in recurrent WHO grade III ependymoma [12, 13] or recurrent WHO grade II and III ependymomas [14]. Activity of TMZ was also described in a retrospective study, analyzing recurrent WHO grade II ependymomas, refractory for platinum-based chemotherapy [15], as well as in a prospective phase II study in ependymoma patients treated with TMZ and lapatinib at tumor recurrence [16]. Brandes and colleagues compared patients with recurrent WHO grade II ependymomas treated with cisplatin-based CT or treated with another kind of CT, demonstrating no significant differences regarding PFS or OS [17]. Here we performed a retrospective analysis of outcome data in 17 adult patients diagnosed with intracranial WHO grade II or III ependymoma and treated with chemotherapy at any time during their disease course.

## Methods

### Patients and tumors

In accordance with approval from the appropriate Institutional Review Boards, the surgical specimens and clinical records were retrieved from 17 patients treated at the University Hospital Zurich, Zurich, Switzerland, or at one of the eight University Hospitals in Germany participating in the German Glioma Network (GGN) (http://www.gliomnetzwerk.de). All patients gave written informed consent according to the research proposals approved by the Institutional Review Boards of the participating institutions (University of Zurich, Switzerland; Universities of Bochum, Bonn, Dresden, Düsseldorf, Hamburg, Heidelberg, Munich and Tübingen, all Germany). All tumors were classified and graded according to the WHO classification of tumors of the central nervous system [4]. The *O*^*6*^*-methylguanyl-DNA-methyltransferase (MGMT)* promoter methylation status was determined by methylation-specific polymerase chain reaction in 12 tumors [18]. Epidemiological and treatment data were taken from patient health records. Radiological response rates to chemotherapy were documented using Macdonald criteria [19] as foreseen in the German Glioma Network study protocol.

### Statistics

PFS and OS curves were estimated by the Kaplan-Meier method. PFS was calculated from the date of first chemotherapy to the date of progression. OS was measured from the date of first chemotherapy to the date of death. Patients without confirmed death were censored for OS at the last follow-up visit. Patients without documented progression were censored at the last follow-up visit for PFS. Survival-related analyses were calculated with the log-rank test. A cox proportional hazard model was used for univariate analysis, to test the association of clinical predictors with survival outcomes from start of chemotherapy. All statistical tests were two-tailed, and a *p* value of 0.05 was set as statistically significant. All statistical analyses were performed using Prism 6 (GraphPad Software) or Statistics 22 (SPSS software).

## Results

### Patient characteristics

Table 1 summarizes the principle patient characteristics: 17 patients initially diagnosed with intracranial ependymoma WHO grade II or III were studied. Median age at diagnosis was 28 years (range 18–56 years, mean age 33 years, 95 % confidence interval (CI) 27–40); 14 patients were men (82.4 %); 11 patients had supratentorial (64.7 %) and 6 patients had infratentorial (35.3 %) tumors. Most patients (58.8 %) had a Karnofsky Performance Score (KPS) of 90–100 % at the time of initial diagnosis. At time of first chemotherapy, most patients (52.9 %) had a KPS of 70–80 %. Histology at time of first surgical intervention revealed ependymoma WHO grade II in 4 patients and anaplastic ependymoma WHO grade III in 13 patients. Nine patients were treated with RT alone (52.9 %) as first-line therapy post-surgery, 2 patients with anaplastic ependymoma received radiotherapy plus concomitant and maintenance temozolomide chemotherapy (TMZ/RT → TMZ) [20] at this timepoint, while 6 patients did not receive upfront treatment. At recurrence, 11 patients (64.7 %) underwent another surgical tumor resection and 6 patients (35.3 %) received CT treatment at this time. The remaining 9 patients received the first CT treatment at subsequent recurrences. In all cases tumor recurrence was local when CT was started. Three patients (patients 7, 8 and 14) experienced spinal drop metastases later on in the course of disease. Eight patients (47.1 %) were alive at a median follow-up period of 87 months. Median follow-up, defined from start of first chemotherapy, was 39 months for the whole patient group. Individual patient profiles are summarized in Table 2.

**Table 1:** Summary of patient characteristics

	n = 17 patients
Age (years)	
Median	28
Range	18–56
Age classes, n (%)	
≤ 20 years	2 (11.8)
21–30 years	8 (47.1)
31–40 years	0
41–50 years	5 (29.4)
> 50 years	2 (11.8)
Gender	
Female	3 (17.6)
Male	14 (82.4)
KPS (pre-operative), n (%)	
90–100	10 (58.8)
70–80	6 (35.3)
< 70	0
No data	1 (5.9)
KPS (start first CT), n (%)	
90–100	3 (17.6)
70–80	13 (76.5)
< 70	0
No data	1 (5.9)
Tumor localization^c^	
Supratentorial	11 (64.7)
Infratentorial	6 (35.3)
Extent of first resection, n (%)	
Gross total resection	4 (23.5)
Subtotal resection	5 (29.4)
Partial resection	5 (29.4)
Biopsy	2 (11.8)
No data	1 (5.9)
Histology (initial)	
Ependymoma WHO grade II	4 (23.5)
Anaplastic ependymoma WHO grade III	13 (76.5)
Histology (start first CT)	
Ependymoma WHO grade II	1 (5.9)
Anaplastic ependymoma	14 (82.4)
Sarcoma/Gliosarcoma	2 (11.8)
*MGMT* promoter methylation status, n (%)	
Unmethylated	9 (52.9)
Methylated	3 (17.6)
No data	5 (29.1)
First-line therapies beyond surgery, n (%)	
RT alone	9 (52.9)
TMZ/RT → TMZ	2 (11.8)
No therapy	6 (35.3)
First salvage therapy, n (%)	
Re-resection	
alone	4 (23.5)
plus RT alone	2 (11.8)
plus RT plus TMZ	2 (11.8)
plus Bevacizumab	1 (5.9)
plus CT^a^	2 (11.8)
CT alone^b^	4 (23.5)
RT alone	1 (5.9)
Bevacizumab alone	1 (5.9)
First CT, n (%)	
TMZ	10 (58.8)
Procarbazine plus Lomustine plus Vincristine	3 (17.6)
Epirubicin plus Ifosfamide	1 (5.9)
Carboplatin plus Etoposide	2 (11.8)
Carboplatin plus Etoposide plus Vincristine	1 (5.9)
Number of surgical interventions	
1	5 (29.4)
2	3 (17.6)
> 3	9 (52.9)
Survival (from first CT) (all patients n = 17)	
Median follow-up (months)	39
Median PFS (months) (95% CI) (events)	10 (3.4–16.6) (15)
Median OS (months) (95% CI) (events)	41 (31.6–50.4) (9)
Survival (from first CT) (n = 15, patients 5 and 9 excluded^d^)	
Median follow-up (months)	39
Median PFS (months) (95 % CI) (events)	6 (1.5–10.5) (14)
Median OS (months) (95 % CI) (events)	41 (30.0–52) (8)

*CI* confidence interval, *CT* chemotherapy, *KPS* Karnofsky Performance Score, *MGMT* O^6^-methylguanyl-DNA-methyltransferase, *n.a.* not applicable, *OS* overall survival, *PCV* procarbazine/lomustine/vincristine, *PFS* progression-free survival, *RT* radiotherapy, *TMZ* temozolomide

^a^, 1 PCV, 1 TMZ; ^b^, 1 PCV, 2 TMZ, 1 carboplatin plus etoposide; ^c^, tumor localization was the same at date of diagnosis and start of CT; ^d^, patients 5 and 9 were diagnosed with sarcoma or gliosarcoma at recurrence

**Table 2:** Individual patient characteristics^c^

No.	Initial histology	WHO grade	*MGMT* status	Localization	Extent of resection	First-line therapy	First salvage therapy	Surgical interventions (n)	Histology at start of CT	First CT	Duration of first CT ^a^	Best response	DBR^a^	PFS^a^	OS^a^
P1	E	II	unmethyl.	infratentorial	subtotal	-	RT	3	AE	TMZ	11	SD	11	17	68^b^
				IV ventricle											
P2	E	II	unmethyl.	supratentorial	partial	RT	re-resection RT	>3	AE	PCV	18	SD	4	21	41
				parietal											
P3	E	II	unmethyl.	infratentorial	partial	-	re-resection	2	AE	TMZ/RT→TMZ	8	SD	29	32^b^	32^b^
				cerebellar			TMZ/RT→TMZ								
				brainstem											
				IV ventricle											
P4	E	II	unmethyl.	supratentorial	partial	-	re-resection	3	E	TMZ	6	SD	2	5	5
				III ventricle											
P5	AE	III	n.d.	supratentorial	gross total	RT	re-resection	2	sarcoma	epirubicin	5	CR	106	131^b^	131^b^
				parietal						ifosfamide					
				temporal											
				occipital											
P6	AE	III	unmethyl.	infratentorial	subtotal	RT	carboplatin	1	AE	carboplatin	5	PR	7	11	16^b^
				IV ventricle			etoposide			etoposide					
P7	AE	III	n.d.	supratentorial	partial	-	re-resection	>3	AE	carboplatin	1	PD	-	2	44^b^
				parietal						etoposide					
										vincristine					
										cyclo-phosphamide					
P8	AE	III	unmethyl.	infratentorial	gross	-	re-resection	>3	AE	TMZ	17	SD	21	25	80^b^
				IV ventricle	total		RT								
P9	AE	III	n.d.	supratentorial	biopsy	RT	re-resection	3	gliosarcoma	TMZ	9	SD	7	10	23
				frontal			TMZ								
				temporal											
P10	AE	III	n.d.	infratentorial	gross	RT	TMZ	1	AE	TMZ	5	SD	31	33	101^b^
				cerebellar	total										
P11	AE	III	unmethyl.	supratentorial	subtotal	RT	TMZ	1	AE	TMZ	9	SD	9	10	13
				III ventricle											
P12	AE	III	n.d.	supratentorial	subtotal	TMZ/RT → TMZ	re-resection	>3	AE	TMZ/RT → TMZ	5	SD	4	5	48
				parietal			bevacizumab								
P13	AE	III	unmethyl.	supratentorial	gross total	RT	re-resection	3	AE	PCV	8	SD	1	5	40
				parietal			PCV								
P14	AE	III	unmethyl.	supratentorial	biopsy	-	re-resection	2	AE	TMZ	1	PD	-	2	2
				temporal			RT								
				III ventricle			TMZ								
P15	AE	III	methyl.	infratentorial	partial	RT	re-resection	3	AE	carboplatin	n.d.	PD	-	4	29^b^
				cerebellar						etoposide					
				brainstem											
				IV ventricle											
P16	AE	III	methyl.	supratentorial	n.d.	RT	PCV	1	AE	PCV	3	SD	2	6	39
				temporal											
P17	AE	III	methyl.	supratentorial	subtotal	TMZ/RT → TMZ	bevacizumab	1	AE	TMZ/RT → TMZ	1	PD	-	1	6
				parietal											

*AE* anaplastic ependymoma, *CR* complete response, *DBR* duration best response, *E* ependymoma WHO grade II; unmethyl., unmethylated, *MGMT status O* ^*6*^ *-methylguanyl-DNA-methyltransferase* promoter methylation status, *n.d.* no data, *no.* number, *OS* overall survival, *P* patient, *PCV* procarbazine/lomustine/vincristine, *PD* progressive disease, *PFS* progression-free survival, *PR* partial response, *RT* radiotherapy, *SD* stable disease, *TMZ* temozolomide

^a^ in months; ^b^ indicates patients who did not demonstrate progressive disease decease or who were not deceased, ^c^ median age was 28 years (range 18–56), 3 female and 14 male patients were included

At time of recurrence 2 patients initially diagnosed with anaplastic ependymoma were diagnosed with sarcoma (patient 5) or gliosarcoma (patient 9) (Table 2). Both patients had been previously treated with RT. At recurrence patient 5 was treated with 7 cycles of epirubicin/ifosfamide and demonstrated a complete response (CR) with no evidence of recurrence during follow-up of 8.8 years. Patient 9 was treated with 9 cycles of TMZ, best response was stable disease (SD) with PFS of 10 months and OS of 23 months.

### Benefit from chemotherapy

**Fig. 1:**
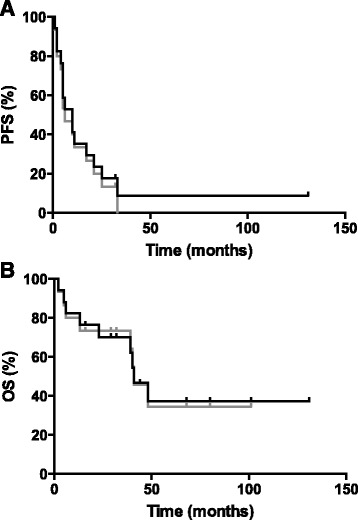
*Outcome in ependymoma patients*. Kaplan-Meier survival curves of PFS (**a**) and OS (**b**) after chemotherapy are shown for all 17 patients, initially diagnosed with ependymoma (black line) or for the 15 patients, diagnosed with ependymoma at time of start CT (grey line); the two patients excluded for the second analysis were initially diagnosed with ependymoma, but diagnosed with sarcoma or gliosarcoma at time of recurrence

At time of first chemotherapeutic treatment, 10 patients were treated with TMZ (58.8 %), 3 patients with procarbazine/lomustine/vincristine (PCV) (17.6 %), 3 patients with platinum-based CT (carboplatin/etoposide (11.8 %) or carboplatin/etoposide/vincristine (5.9 %)) and 1 patient with epirubicin/ifosfamide (5.9 %) (Table 1). Two patients received CT as first line therapy, 8 patients at time of first recurrence and 7 patients at time of second recurrence (Table 2). Median PFS after first chemotherapeutic treatment was 10 months (95 % CI 3.4–16.6) for all patients pooled, as it was for the group of patients treated with TMZ (95 % CI 2.4–17.6); it was 4 (95 % CI 0.8–7.2) months for patients treated with platinum-based CT and 6 months (95 % CI 4.4–7.6) for patients treated with PCV. PFS for the patient treated with epirubicin/ifosfamide was not reached during a follow-up period of 131 months. Median OS from start of chemotherapy was 41 months (95 % CI 31.6–50.4) for all patients pooled, 23 months (95 % CI 0–71.2) for patients treated with TMZ and 40 months (95 % CI 38.4–41.6) for patients treated with PCV. OS in the group of patients treated with platinum-based CT was not reached during the follow-up period. Since two patients (patients 5 and 9), treated with epirubicin/ifosfamide or TMZ, were not diagnosed with classical ependymoma at time of start of chemotherapy, survival data were also assessed for the remaining 15 patients: median PFS after first chemotherapeutic treatment was 6 months (95 % CI 1.5–10.5) for all patients pooled (*n* = 15), and 10 months (95 % CI 0.0–24.6) for patients treated with TMZ; median OS after first chemotherapeutic treatment was 41 months (95 % CI 30.0–52) for all patients pooled (*n* = 15), and 48 months (95 % CI 0.0–133.4) for patients treated with TMZ. In the group of patients treated with TMZ, 8 patients showed SD and 2 patients showed progressive disease (PD). In the group of patients treated with PCV, all 3 patients had SD and among the 3 patients treated with platinum-based CT, 1 patient demonstrated partial response (PR) and 2 patients PD. The patient who received epirubicin/ifosfamide (patient 5) demonstrated CR (Table 2). PFS and OS for all patients are shown in Fig. 1. PFS rates at 6, 12 and 24 months were 52.9, 35.3 and 23.5 % for all 17 patients included in this study, and 46.7, 33.3 and 33.3 % when the two patients diagnosed with sarcoma or gliosarcoma at time of recurrence were excluded. OS rates at 6, 12 and 24 months were 82.4, 82.4 and 70.1 % for all patients, and 80.0, 80.0 and 73.3 % for the reduced patient cohort (*n* = 15). Group sizes were too small for formal comparisons, but not obvious signal of activity of a particular regimen became apparent.

### *MGMT* promoter methylation and survival in intracranial ependymoma

The *MGMT* promoter methylation status of the tumor was available in 12 patients (Table 2): 3 patients had *MGMT* promoter methylated tumors, whereas 9 patients did not. Patients with *MGMT* promoter methylated tumors were treated with TMZ, platinum-based CT or PCV. Patients without *MGMT* promoter methylation were treated with TMZ (6 patients), platinum-based CT (1 patient) or PCV (2 patients). Median PFS was 4 months for patients with *MGMT* promoter methylated tumors and 11 months for patients with *MGMT* promoter unmethylated tumors. Median OS was 39 months for patients with *MGMT* promoter methylated tumors versus 40 months for patients with *MGMT* promoter unmethylated tumors. Group sizes were too small for formal comparisons between patients stratified according to the *MGMT* promoter methylation status.

### Association of age, tumor localization, gender, KPS and *MGMT* promoter methylation status with survival

Patients were divided into two groups, defined by age, tumor localization, gender, KPS and *MGMT* promoter methylation status, and a log-rank survival analysis was performed. This analysis revealed supratentorial tumors to be associated with inferior survival (Table 3, Additional file 1: Table S1). Univariate analysis using the cox proportional hazard model from start of first CT treatment was performed to identify factors associated with overall survival. The results of these analyses are summarized in Table 3 for all 17 patients initially diagnosed with ependymoma, and in Additional file 1: Table S1 for the remaining patient cohort when the two patients diagnosed with sarcoma or glioblastoma at recurrence were excluded. Age, tumor localization, gender, KPS and the *MGMT* promoter methylation status were not identified as risk factors for death in both patient cohorts (Table 3, Additional file 1: Table S1).

**Table 3:** Association of age, tumor localization, gender, KPS and *MGMT* promoter methylation status with survival (all patients, *n* = 17)

Variable	Number of patients (events)	Median OS (months) (95 % CI)	*p* value (log-rank)	*p* value (cox regression)	Hazard Ratio (95 % CI)
Age^a^					
< 40 years	9 (5)	48 (28.5–67.5)			1
≥ 40 years	8 (4)	41 (13.8–68.14)	0.587	0.589	1.45 (0.37–5.64)
Tumor localization^a^					
Infratentorial	6 (0)	undefined			1
Supratentorial	11 (9)	39 (9.87–68.13)	0.011 (*)	0.163	50.8 (0.20–12662.82)
Gender					
Female	3 (2)	41 (0–97.01)			1
Male	14 (7)	48 (35.84–60.16)	0.888	0.888	0.89 (0.18–4.34)
KPS^a^					
100–80	11 (6)	48 (14.47–81.54)			1
< 80	5 (3)	41 (11.96–70.04)	0.872	0.872	1.12 (0.28–4.52)
MGMT promoter methylation					
not methylated	9 (5)	40 (9.3–70.7)			1
methylated	3 (2)	39 (undefined)	0.433	0.443	2.02 (0.34–12.2)

*CI* confidence interval, *KPS* Karnofsky Performance Score, *WHO* World Health Organisation

*, *p* < 0.05

^a^, variables were determined at start of chemotherapy

## Discussion

Intracranial ependymoma is a rare disease in adults [2]. While the role of surgical resection and RT for tumor control is undisputed [2, 3, 6–9, 11], little is known about the significance of CT. Here we retrospectively analyzed 17 adult patients with intracranial WHO grade II or grade III ependymomas to investigate the impact of chemotherapy on the disease course.

Most patients (*n* = 10) received TMZ as their first chemotherapeutic treatment (Table 1). Two of these patients received a combined treatment of irradiation and TMZ (TMZ/RT → TMZ) [20] at diagnosis. Median PFS for these two patients was low with 3 months as it was for median OS with 27 months. The other 8 patients were treated with TMZ at recurrence and the best response was SD. Thus, TMZ demonstrated some activity in intracranial ependymoma patients, especially at time of recurrence. Of note, there is one prospective study presented at the American Society of Clinical Oncology (ASCO) in 2005, which included patients with recurrent WHO grade II and III ependymomas. Activity of TMZ treatment in these patients was as follows (best response: median PFS): CR (*n* = 2): 9–48 months; PR (*n* = 3): 4–15 months; SD (*n* = 5): 7–44 months [14] (Table 4). Less optimal results were reported by Chamberlain and Johnston for TMZ treatment of 25 patients with recurrent WHO grade II ependymomas, refractory to platinum-based chemotherapy, who demonstrated a median PFS of 3 months, and best responses of PR in 1 patients and SD in 9 patients with a median OS of 3 months [15] (Table 4). Moreover, results of a prospective trial, including 24 patients diagnosed with WHO grade II ependymoma and 18 patients diagnosed with anaplastic ependymoma, treated with TMZ plus lapatinib, were presented at the annual meeting of the Society of Neurooncology (SNO) in 2014 [16]. Lapatinib targets the epidermal growth factor receptor (ErbB1) and the related family member HER-2/neu (ErbB2) on the cell surface of the tumor cells. Median PFS was 45 weeks for patients diagnosed with grade II, and 25.3 weeks for patients diagnosed with grade III ependymomas. Best response rates were CR in one patient diagnosed with anaplastic astrocytoma and PR in 1 patient diagnosed with anaplastic astrocytoma and 2 patients diagnosed for ependymoma WHO grade II. Several patients showed at least SD. First results of a molecular classification revealed a correlation of response with ErbB2 expression [16]. In addition, there are two case reports of patients diagnosed with recurrent anaplastic ependymoma, describing a median PFS after TMZ treatment of 5 and 9 months [12, 13] (Table 4). One reason for the possible failure of TMZ in patients with intracranial glioma is a non-methylated *MGMT* promoter [21]. Ependymomas may express high levels of MGMT [22], predicting less benefit from TMZ-based CT. Only 3 out of 12 ependymoma patients included in our study had tumors with a methylated *MGMT* promoter. Median PFS in these 3 patients was in the range of 1–6 months and thus low, and one of these patients treated with TMZ demonstrated no response. Our small dataset fails to indicate a favorable prognostic role of *MGMT* promoter methylation in adult ependymoma.

**Table 4:** Review of the literature: chemotherapeutic treatment regimens in adult intracranial ependymoma

First author, year [Ref]	Trial design	Patient diagnosis, n	Tumor localization	Treatment regimen, n	Response rates, n	Median PFS, after start CT	Median OS, after start CT
present study	Retrospective	newly diagnosed and recurrent WHO grade II and III ependymoma, 17	supratentorial or infratentorial	TMZ-based CT: 10	CR: 1	10 months	41 months
				platinum-based CT: 3	PR: 1		
				PCV: 3	SD: 11		
				epirubicin/ifosfamide: 1 (no prior CT)	PD: 4		
Gilbert et al., 2014 [16]	Prospective	recurrent WHO grade II ependymoma, 24 and grade III,18 ependymoma	intracranial and/or spinal	TMZ plus lapatinib	WHO grade II	WHO-grade II: 45 weeks	-
					PR: 2	WHO-grade III: 25.3 weeks	
					SD/PD: no data		
					WHO grade III		
					CR: 1		
					PR: 1		
					SD/PD: no data		
Chamberlain and Johnston, 2009 [15]	Retrospective	recurrent WHO grade II ependymoma, 25	supratentorial	TMZ (after platinum-based CT)	PR: 1	2 months	3 months
					SD: 9		
					PD: 15		
Green et al., 2009 [26]	Retrospective	recurrent WHO grade II and III ependymoma, 8	supratentorial or infratentorial	bevacizumab (after platinum-based CT or TMZ)	PR: 6	6.4 months	9.4 months
					SD: 1		
					PD: 1		
Brandes et al., 2005 [17]	Retrospective	recurrent WHO grade II and III ependymoma, 28	intracranial	cisplatin-based CT: 13 (no prior CT)	CR: 2	9.9 months	31 months
					PR: 2		
					SD: 7		
					PD: 2		
				versus	versus	versus	versus
				CT without cisplatin: 15 (no prior CT)	PR: 2	10.9 months	40.7 months
					SD: 11		
					PD: 2		
Soffietti et al., 2005 [14]	Prospective	recurrent WHO grade II and III ependymoma, 11	intracranial	TMZ (some after nitrosourea or platinum-based CT)	CR: 2	CR: 9–48+ months	-
					PR: 3	PR: 4–15+ months	
					SD: 5	SD: 7–44+ months	
					PD: 1		
Lombardi et al., 2013 [13]	case report	recurrent anaplastic ependymoma	supratentorial	TMZ plus cisplatin (after platinum-based CT alone and TMZ alone)	PR	9 months	11 months
Freyschlag et al., 2011 [12]	case report	recurrent anaplastic ependymoma	supratentorial	TMZ	no evidence of radiographic progression	5+ months	-
Rojas-Marcos et al., 2003 [25]	case report	recurrent anaplastic ependymoma	infratentorial (initial) and supratentorial (at recurrence)	tamoxifen plus isotretinoin (after TMZ, platinum-based CT, CCNU)	CR	17 months	-

*CR* complete response, *CT* chemotherapy, *n* number of patients, *OS* overall survival, *PCV* procarbazine/lomustine/vincristine, *PD* progressive disease, *PFS* progression-free survival, *PR* partial response, *SD* stable disease, *TMZ* temozolomide

+ indicates patients who did not demonstrate progressive disease

The other chemotherapeutic regimens were platinum-based CT (*n* = 3) or PCV (*n* = 3), showing a median PFS of 4 or 6 months and response rates as follows: PR (1 patient) or SD (3 patients) (Table 2). Brandes et al. published a retrospective study in 2005, analyzing 28 ependymoma patients (WHO grade II and III); 13 patients were treated with platinum-based CT, demonstrating best responses of 2 CR, 2 PR and 7 SD, and a median PFS of 9.9 months in comparison to 15 patients treated with CT without cisplatin, demonstrating best responses of 2 PR and 11 SD, and a median PFS of 10.9 months [17]. PFS data were similar to the data reported here. Although response rates were lower in the group of patients treated with any other CT, but without cisplatin, survival curves (PFS and OS) did not differ. Three of the 15 patients were treated with PCV, showing best responses with 1 PR, and 2 SD, therefore demonstrating a similar response as reported here (Tables 2 and 4).

Beyond these mentioned standard chemotherapeutic drugs, there is need for patient stratification based on molecular markers to identify ependymoma subgroups as well as subgroup-specific therapies, as recently described by Pajtler et al. [23]. Since all pre-clinical in vivo models tested for chemotherapy so far showed only reduced sensitivity towards these standard chemotherapeutic drugs, targeted therapies (e.g. epidermal growth factor receptor (EGFR) inhibitors, histone deacetylase (HDAC) inhibitors) are in the focus of research at the moment and need to be investigated [24].

Review of the literature showed a case report demonstrating a CR and a median PFS of 17 months in an adult patient with recurrent anaplastic intracranial ependymoma after treatment with tamoxifen and isotretinoin [25]. Beyond classical CT, Green et al. reported outcome data in 8 recurrent WHO grade II and III intracranial ependymomas which were treated with bevacizumab at time of recurrence. Promising response rates with 6 PR and 1 SD and a median PFS of 6.4 and OS of 9.4 months were described [26].

Two patients in our study were initially diagnosed with anaplastic ependymoma and at recurrence demonstrated sarcoma or gliosarcoma (Table 2). Both patients had been treated with RT in-between and had supratentorial tumors. A common origin for the initial and the recurrent tumor has to be considered, since there are reports in the literature demonstrating identical genetic mutations in both glial and sarcomatous compartments of gliosarcomas [16, 27, 28]. Interestingly, both patients appeared to derive benefit from chemotherapy.

Kaplan-Meier survival analysis revealed supratentorial tumor location as a parameter associated with inferior survival (Table 3). Supratentorial tumor localization has been described as significantly increasing risk of early death [29], therefore underlining the clinical aggressiveness of supratentorial ependymomas in adults that is independent from CT treatment. Our data suggest that survival outcomes in response to chemotherapy in adult intracranial ependymoma patients vary substantially, but individual patients may respond to any kind of chemotherapy.

## Conclusions

The main limitation of our study is its retrospective design and the low number of patients in each chemotherapeutic subgroup. In summary, this retrospective study provides data supporting activity of TMZ in recurrent anaplastic ependymoma; however, there are also promising response rates in patients treated with platinum-based CT or PCV. Because of the notably individual survival outcomes after chemotherapeutic treatment in adult ependymoma patients with intracranial disease, prospective studies are urgently needed to identify patient subgroups that will benefit from individual chemotherapeutic treatments. Yet, this report suggests that at least one line of CT should be offered to ependymoma patients who are no longer candidates for surgery or RT.

### Availability of data and materials

The dataset supporting the conclusions of this article is included within the article in Table 2. Full data on all patients are available in the database of the German Glioma Network (GGN) (http://www.gliomnetzwerk.de), Leipzig, Germany, that is not open for public. Data on patients from the University Hospital Zurich are available at a local database of the Department of Neurology, Zurich, Switzerland, that is also not open for the public.

## Additional file

Additional file 1: Table S1 Association of age, tumor localization, gender, KPS and MGMT promoter methylation status with survival (*n* = 15, patients 5 and 9 excluded°). (PDF 82 kb)

## Abbreviations

ASCO: American Society of Clinical Oncology
CI: confidence interval
CR: complete response
CT: chemotherapy
EGFR: epidermal growth factor receptor
GGN: German Glioma Network
HDAC: histone deacetylase
KPS: Karnofsky Performance Score
MGMT: O^6^-methylguanyl-DNA-methyltransferase
OS: overall survival
PCV: procarbazine/lomustine/vincristine
PD: progressive disease
PFS: progression-free survival
PR: partial response
RT: radiotherapy
SD: stable disease
SNO: Society of Neurooncology
TMZ: temozolomide, TMZ/RT → TMZ, radiotherapy plus concomitant and maintenance temozolomide chemotherapy
WHO: World Health Organisation
