# Supplementary material for: Chemotherapy for intracranial ependymoma in adults
Source: BMC Cancer. 2016 Apr 23;16:287. doi: 10.1186/s12885-016-2323-0 (PMC4842281; doi:10.1186/s12885-016-2323-0)
Supplement: Additional file 1: Table S1 — Association of age, tumor localization, gender, KPS and MGMT promoter methylation status with survival (n = 15, patients 5 and 9 excluded°). (PDF 82 kb) [file 12885_2016_2323_MOESM1_ESM.pdf]

Additional File

Chemotherapy for intracranial ependymoma in adults

Dorothee Gramatzki[1], Patrick Roth[1], Jörg Felsberg[2], Silvia Hofer[3], Elisabeth J. Rushing[4], Bettina Hentschel[5], Manfred Westphal[6], Dietmar Krex [7], Matthias Simon[8], Oliver Schnell[9], Wolfgang Wick[10,11], Guido Reifenberger[12], Michael Weller[1,11]

[1]Department of Neurology, University Hospital Zurich, Frauenklinikstrasse 26, 8091 Zurich, Switzerland

[2]Department of Neuropathology, Heinrich-Heine-University, Moorenstrasse 5, 40225 Düsseldorf, and German Cancer Consortium (DKTK), partner site Essen/Düsseldorf, German Cancer Research Center (DKFZ), Im Neuenheimer Feld 280, 69121 Heidelberg, German

[3]Department of Oncology, University Hospital Zurich, Rämistrasse 100, 8091 Zurich, Switzerland

[4]Department of Neuropathology, University Hospital Zurich, Schmelzbergstrasse 12, 8091 Zurich, Switzerland

[5]Institute for Medical Informatics, Statistics and Epidemiology, University of Leipzig, Härtelstrasse 16-18, 04107 Leipzig, Germany

[6]Department of Neurosurgery, University Medical Center Hamburg-Eppendorf, Martinistrasse 52, 20251 Hamburg, Germany

[7]Department of Neurosurgery, Technical University Dresden, Fetscherstrasse 74, 01307 Dresden, Germany

[8]Department of Neurosurgery, University of Bonn Medical School, Sigmund-Freud-Strasse 25, 53127 Bonn, Germany

[9]Department of Neurosurgery, Ludwig Maximilian University Munich, Marchionistrasse 15, 81377 Munich, Germany

[10]Clinical Cooperation Unit Neurooncology, German Cancer Consortium (DKTK), German Cancer Research Center (DKFZ), and Neurology Clinic and National Center for Tumor Diseases, University Hospital Heidelberg, Im Neuenheimer Feld 672, 69120 Heidelberg, Germany

[11]Department of General Neurology, University Hospital Tübingen, Hoppe-Seyler-Strasse 3, 72076 Tübingen, Germany

Table S1. Association of age, tumor localization, gender, KPS and *MGMT* promoter methylation status with survival (n=15, patients 5 and 9 excluded°).

Variable	Number of patients (events)	Median OS (months) (95% CI)	*p* value (log-rank)	*p* value (cox regression)	Hazard Ratio (95% CI)
Age⁺					
< 40 years	8 (5)	40 (29.3 – 50.7)			1
≥ 40 years	7 (3)	41 (0.00 – 92.2)	0.978	0.978	1.02 (0.24 – 4.4)
Tumor localization⁺					
Infratentorial	6 (0)	undefined			1
Supratentorial	9 (8)	39 (0.00 – 114.97)	0.005 (*)	0.146	71.19 (0.23 – 22477.36)
Gender					
Female	3 (2)	41 (0 – 97.01)			1
Male	12 (6)	40 (29.92 – 50.08)	0.954	0.954	0.95 (0.19 – 4.78)
KPS⁺					
100-80	10 (5)	48 (26.86 – 69.15)			1
< 80	4 (3)	40 (0.00 – 90.9)	0.348	0.357	2.03 (0.45 – 9.19)
MGMT promoter methylation					
not methylated	9 (5)	40 (9.3 – 70.7)			1
methylated	3 (2)	39 (undefined)	0.433	0.443	2.02 (0.34 – 12.2)

CI, confidence interval; KPS, Karnofsky performance score; WHO, World Health Organisation; *, $p < 0.05$; ⁺, variables were determined at start of chemotherapy; °, patients 5 and 9 were diagnosed with sarcoma or gliosarcoma at recurrence.
